# SUCCOR Nodes: May Sentinel Node Biopsy Determine the Need for Adjuvant Treatment?

**DOI:** 10.1245/s10434-023-13529-w

**Published:** 2023-05-19

**Authors:** Arantxa Berasaluce Gómez, Nerea Martín-Calvo, Félix Boria, Nabil Manzour, Enrique Chacón, Nicolò Bizzarri, Luis Chiva, SUCCOR Study Group, Alejandra Martinez, Alfonso Quesada, Ali Kucukmetin, Alicia Vázquez, Aljosa Mandic, Andrea Casajuana, Andreas Kavallaris, Anna Fagotti, Anna Perrone, Annamaria Ferrero, Arantxa Lekuona, Arno Uppin, Artem Stepanyan, Benito Chiofalo, Bibiana Morillas, Carmen Tauste, Claudia Andrade, Constantijne Mom, Cosima Brucker, Cosmin-Paul Sarac, Daniel Vázquez-Vicente, David Cibula, Denis Querleu, Diego Erasun, Dilyara Kaidarova, Dimitrios Tsolakidis, Dimitros Haidopoulos, Dmytro Golub, Eduard-Aexandru Bonci, Elif Aksahin, Elisabete Gonçalves, Enrique Moratalla, Erbil Karaman, Eva Myriokefalitaki, Fabio Ghezzi, Fabrice Narducci, Fernando Roldan, Francesco Raspagliesi, Frédéric Goffin, Frederic Grandjean, Frédéric Guyon, Fuat Demirkiran, Gabriel Fiol, Galina Chakalova, Gemma Mancebo, George Vorgias, Gerhard Gebauer, Gesine Meili, Gines Hernandez-Cortes, Giorgio Bogani, Gloria Cordeiro, Goran Vujić, Gustavo Mendinhos, Hans Trum, Hélène Bonsang-Kitzis, Herman Haller, Ignace Vergote, Ignacio Zapardiel, Igor Aluloski, Igor Berlev, Imre Pete, Ioannis Kalogiannidis, Ioannis Kotsopoulos, Iryna Yezhova, Javier Díez, Jean G. Feron, Jens-Peter Scharf, Jogchum Beltman, Jolien Haesen, Jordi Ponce, Jorge Cea, Jose Ángel Mínguez, José García, Juan Arévalo-Serrano, Juan Gilabert, Juan Luis Alcazar, Kersti Kukk, Khadra Galaal, Laura Cárdenas, Laurentiu Pirtea, Liliana Mereu, Luigi Pedone Anchora, Lukas Dostalek, Lukasz Klasa, Maja PakižImre, Manuela Undurraga, Marcin Jedryka, Margarida Bernardino, Maria Alonso-Espias, María Belén Martín-Salamanca, Maria Cuadra, Mariana Tavares, Mario Malzoni, Mathias Fehr, Mathieu Luyckx, Maximilian Lanner, Meelis Leht, Mehmet Meydanli, Michael Mallmann, Mihai Căpîlna, Mikulás Redecha, Milena Mitrovic, Minna M. Maenpaa, Miriam Guijarro, Nabil Abdalla, Nana Gomes, Natalia Povolotskaya, Nikola Badzakov, Octavio Arencibia, Özgür Akbayir, Pere Cavalle, Petra Zusterzeel, Philip Rolland, Pluvio Coronado, Rasiah Bharathan, Reeli Saaron, Rita Sousa, Robert Fruscio, Robert Jach, Robert Poka, Rosa Barrachina, Santiago Domingo, Sara Morales, Sedat Akgöl, Sergi Fernandez-Gonzalez, Shamistan Aliyev, Sofía Herrero, Soledad Fidalgo, Sonia Prader, Špela Smrkolj, Stamatios Petousis, Stefan Kovachev, Taner Turan, Tayfun Toptas, Teresa Castellanos, Teresa Diniz da Costa, Tiermes Marina, Vanna Zanagnolo, Victor Martin, Virginia Gonzalez, Vladimír Študent, Vladyslav Sukhin

**Affiliations:** 1 Department of Preventive Medicine and Public Health, School of Medicine, University of Navarra, Pamplona, Spain; 2 IdiSNA, Instituto de Investigación Sanitaria de Navarra, Pamplona, Spain; 3 CIBER de Fisiopatología de la Obesidad y la Nutrición, Instituto de Salud Carlos III, Madrid, Spain; 4 Department of Obstetrics and Gynecology, Universidad de Navarra Clinic, Madrid, Spain; 5 Department of Obstetrics and Gynecology, Universidad de Navarra Clinic, Pamplona, Spain; 6 Dipartimento per la Salute della Donna e del Bambino e della Salute Pubblica, Fondazione Policlinico Universitario A. Gemelli, IRCCS, UOC Ginecologia Oncologica, Rome, Italy

## Abstract

**Background:**

The SUCCOR cohort was developed to analyse the overall and disease-free survival at 5 years in women with FIGO 2009 stage IB1 cervical cancer. The aim of this study was to compare the use of adjuvant therapy in these women, depending on the method used to diagnose lymphatic node metastasis.

**Patients and Methods:**

We used data from the SUCCOR cohort, which collected information from 1049 women with FIGO 2009 stage IB1 cervical cancer who were operated on between January 2013 and December 2014 in Europe. We calculated the adjusted proportion of women who received adjuvant therapy depending on the lymph node diagnosis method and compared disease free and overall survival using Cox proportional-hazards regression models. Inverse probability weighting was used to adjust for baseline potential confounders.

**Results:**

The adjusted proportion of women who received adjuvant therapy was 33.8% in the sentinel node biopsy + lymphadenectomy (SNB+LA) group and 44.7% in the LA group (*p* = 0.02), although the proportion of positive nodal status was similar (*p* = 0.30). That difference was greater in women with negative nodal status and positive Sedlis criteria (difference 31.2%, *p* = 0.01). Here, those who underwent a SNB+LA had an increased risk of relapse [hazard ratio (HR) 2.49, 95% confidence interval (CI) 0.98–6.33, *p* = 0.056] and risk of death (HR 3.49, 95% CI 1.04–11.7, *p* = 0.042) compared with those who underwent LA.

**Conclusions:**

Women in this study were less likely to receive adjuvant therapy if their nodal invasion was determined using SNB+LA compared with LA. These results suggest a lack of therapeutic measures available when a negative result is obtained by SNB+LA, which may have an impact on the risk of recurrence and survival.

**Supplementary Information:**

The online version contains supplementary material available at 10.1245/s10434-023-13529-w.

In 2018, 61,000 new cases of cervical cancer were reported in Europe, which accounted for 25,800 new deaths.^1^ Given its high prevalence, efforts are made to ensure the best management of patients to ensure survival and quality of life. Given the advances in technology, diagnostic protocols have undergone modifications given the benefits proven by magnetic resonance imaging (MRI) and positron emission tomography (PET) imaging,^2^ and most importantly, the use of sentinel node biopsy (SNB).

Lymph node invasion is one of the most relevant prognostic factors in cervical cancer.^3^ For this reason, works in investigation have been oriented towards optimising lymphatic diagnosis. So much so, that it is now a recommended standard in clinical practice to add sentinel node biopsy (SNB) to lymphadenectomy to increase precision in this aspect.^4^ Nevertheless, as of now, whether SNB should substitute lymphadenectomy as diagnostic method of lymph node metastasis remains under investigation. Furthermore, it is also unclear how performing SNB may be affecting the management of women with early-stage cervical cancer.

The standard treatment of early-stage cervical cancer, as described in the clinical guidelines, consists primarily of a type C radical hysterectomy, with the possibility of considering a modified radical hysterectomy in selected cases.^4, 5^ In women with negative lymph nodes at imaging, SNB is strongly recommended before pelvic lymphadenectomy.^4^ In patients with unequivocally positive nodal invasion in radiological imaging, definite chemoradiotherapy is recommended, and debulking of suspicious lymph nodes may be considered.^4^

Adjuvant therapy in early-stage cervical cancer has been widely debated in the past years, especially in women without lymph node metastasis. Two randomized clinical trials published by the Gynecologic Oncology Group demonstrated a prolonged overall and disease-free survival in women treated with adjuvant therapy, with a significant cost in morbi-mortality resulting from complications.^6, 7^ As of now, European guidelines state that adjuvant radiotherapy should be considered (though not specifically obliged) in women that, without nodal invasion in the definitive pathological study, present a combination of adverse prognostic factors such as large tumor size, lymphovascular space invasion and deep stromal invasion (known as “intermediate risk” factors).^4^ The 2018 FIGO guidelines, on the other hand, do recommend the use of postoperative radiotherapy without chemotherapy in patients that present intermediate risk factors (tumor size ≥ 4 cm, lymphovascular space invasion and deep stromal invasion).^5^

All in all, the primary objective of this study was to assess the rate of adjuvant therapy in women with FIGO 2009 stage IB1 cervical cancer, whose lymph node status was evaluated using lymphadenectomy compared with SNB + lymphadenectomy. The secondary objective was to compare the risk of relapse and death of the disease in these groups of patients.

## Patients and Methods

### Study Design

SUCCOR is a European multicentre observational retrospective cohort that recruited patients from 1 January 2013 until 31 December 2014, with the goal of analyzing the overall and disease-free survival at 5 years in women with FIGO 2009 stage IB1 cervical cancer (FIGO 2018 IB1 and IB2). All ESGO members were invited to participate in this study. Researchers from 126 institutions in 29 European countries registered and contributed to the project. The study is registered at ClinicalTrials.gov with the identifier NCT03958305. In brief, the SUCCOR study focused on comparing the disease-free survival of women with early-stage cervical cancer who underwent radical hysterectomy by laparotomy versus laparoscopy. Further details of the SUCCOR study can be found elsewhere.^8^ For this study, we used data from women in the SUCCOR cohort to compare the use of adjuvant therapy associated with the method used to assess node metastasis (only lymphadenectomy versus lymphadenectomy + sentinel node biopsy). Inclusion and exclusion criteria are listed in the Supplementary Table 1.

### Group Definition and Outcomes

Women were classified into two groups depending on the lymph node diagnosis method (only lymphadenectomy versus SNB + lymphadenectomy). The primary outcome was the use of adjuvant therapy, either radiotherapy (standard external radiation or intracavitary brachytherapy) or chemoradiation post radical hysterectomy.

In further analyses we compared disease-free survival and overall survival associated with the method of lymph node assessment. Disease-free survival was established as the time from the date of the radical hysterectomy until the time of relapse or last contact, whichever came first. Overall survival was calculated as the time from the radical hysterectomy until the time of last contact or death from cervical cancer, whichever came first.

### Sample Size Calculation

In our primary analysis, for a 3:1 ratio of patients diagnosed using lymphadenectomy versus SNB + lymphadenectomy, we hypothesized that 10% of the women in the group that underwent only lymphadenectomy would relapse. Assuming a two-sided alpha error of 5% and an 80% of statistical power, 546 women were needed in the lymphadenectomy group and 182 women were required in the SNB + lymphadenectomy group to detect a 9% difference in the risk of relapse.

### Statistical Analysis

We used inverse probability weighting (IPW) based on propensity score to construct a weighted cohort that accounted for baseline differences between women in both groups. Propensity scores are constructed to predict an exposure and can be used to control confounding bias in numerous ways. IPW is an example of a propensity score that includes the possible confounder as a covariate in the regression of Y (the outcome) on the exposure. When IPW is used, a model on the regression of Y (the outcome) on X (the exposure) is fitted, in which each subject receives a weight that is the inverse of the probability of getting the exposure the subject actually had^8^ Therefore, this method results in a weighted sample in which the variables included in the propensity score are balanced in exposed and non-exposed subjects. The variables included in the propensity score were surgical approach, use of uterine manipulator, lymphovascular space invasion, parametrial space invasion, and conization. The same stabilized weights were used in all the analyses. Missing data were accounted for by group-specific median imputation for quantitative variables and categorization for categorical ones.

For descriptive purposes, we used the mean (standard deviation) for quantitative variables and proportions for categorical ones. Student’s *t*-test and chi-squared test were used for between group comparison of quantitative and categorical variables, respectively.

In the main analysis, we used weighted logistic regression models to calculate the adjusted proportion and 95% confidence interval (CI) of women that received adjuvant therapy in each group. Additionally, we performed subgroup analyses restricted to women with negative nodal status and stratified by their compliance with Sedlis criteria (Supplementary Table 2).

In further analyses we calculated the marginal effect of lymph node diagnosis method on the incidence rate of relapse and death of the disease in the group of women with negative nodal status and positive Sedlis criteria. The 95% confidence limits were the 2.5th and 97.5th percentiles of the distribution obtained from a nonparametric bootstrap with 2000 samples. Weighted Cox proportional-hazards regression models were performed to calculate the hazard ratio (HR) and 95% CI for disease-free survival and overall survival associated with the lymph node diagnosis method in that subgroup of women using the lymphadenectomy group as reference category. The results were plotted using weighted Nelson–Aalen incidence curves.

In sensitivity analyses we repeated the main analysis after excluding women in the SNB + lymphadenectomy group for whom the information about the lymphadenectomy was not clear. All analysis were performed using STATA16, and *p*-values below 0.05 were considered statistically significant.

## Results

**Fig. 1:**
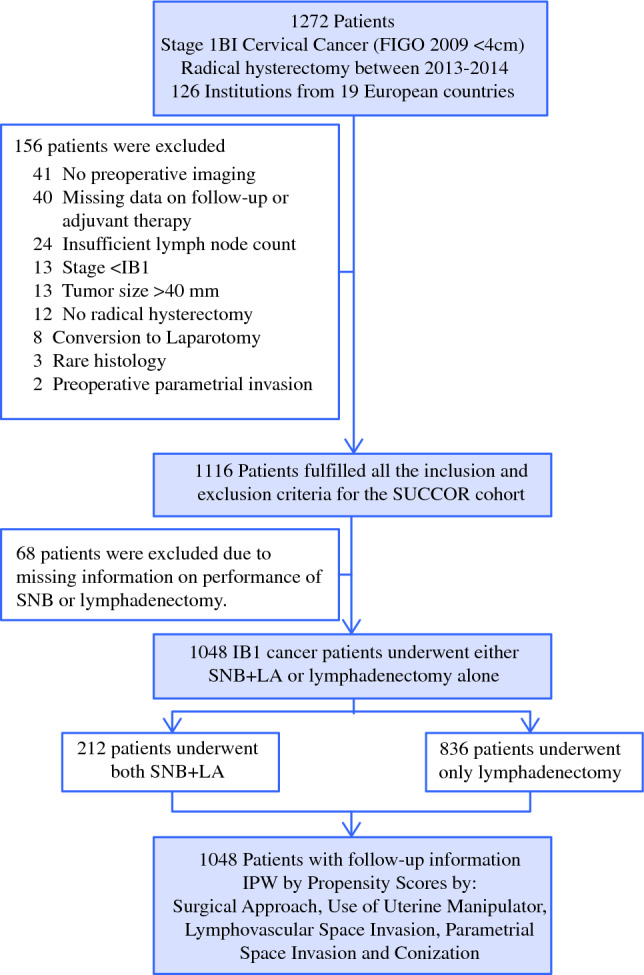
Flowchart of participants. *SNB* sentinel node biopsy, *LA* lymphadenectomy

The initial sample included 1272 women who underwent a radical hysterectomy for FIGO 2009 stage IB1 cervical cancer. From these, 156 patients were excluded either for non-compliance with inclusion–exclusion criteria, or for missing information regarding their status at follow-up, and 68 women were excluded for missing information on method of lymph node assessment. All in all, information from 1048 women was analyzed for the primary endpoint (Fig. 1).

Among the 1048 women included in this study, 836 (79.7%) only received a lymphadenectomy, whereas 212 (20.2%) underwent SNB + lymphadenectomy. Patients diagnosed using SNB + lymphadenectomy were more likely operated through laparoscopy and using uterine manipulator than patients in the lymphadenectomy group. They were also more likely to have had a conization performed, but were less likely to receive protective meneuvers. After inverse probability weighting, no significant differences were observed between both groups (Table 1).

**Table 1:** Sociodemographic and tumor-related characteristics at the time of diagnosis for women in the SUCCOR cohort by lymph node metastasis method of diagnosis [Sentinel node biopsy (SNB) and lymphadenectomy or lymphadenectomy]

	Initial cohort			Weighted cohort		
	SNB + lymphadenectomy	Lymphadenectomy	*p*	SNB + lymphadenectomy	Lymphadenectomy	*p*
	*N* = 212	*N* = 836		*N* = 212	*N* = 836	
	Mean (SD) or *N* (%)			Mean (SD) or *N *(%)		
Age (years)	46.75 (10.44)	47.20 (10.93)	0.576	45.81 (10.08)	46.98 (10.99)	0.162
Age			0.600			0.097
< 50 years old	135 (63.7)	516 (61.7)		69.6	62.3	
≥ 50 years old	77 (36.3)	320 (38.3)		30.4	37.7	
Body mass index (kg/m^2^)	24.88 (4.63)	25.38 (4.27)	0.160	24.77 (4.12)	25.35 (4.28)	0.076
BMI			0.772			0.915
BMI ≤ 25	113 (53.3)	394 (47.1)		51.9	47.0	
BMI > 25	79 (37.3)	289 (34.6)		37.3	34.6	
Not reported	20 (9.4)	153 (18.3)		10.8	18.4	
ECOG score			0.796			0.826
ECOG = 0	194 (91.5)	756 (90.4)		90.9	90.6	
ECOG = 1	13 (6.1)	55 (6.6)		5.8	6.2	
Not reported	5 (2.4)	25 (3.0)		3.3	3.2	
Smoker			0.869			0.529
Non smoker	136 (64.2)	462 (55.3)		58.0	55.4	
Smoker	48 (22.6)	158 (18.9)		23.2	19.0	
Not reported	28 (13.2)	216 (25.8)		18.7	25.6	
Tumor volume (mm^3^)	7422.10 (9612.64)	8010.72 (11,680.97)	0.447	8361.41 (10,590.48)	7814.94 (11,371.76)	0.528
Diameter in AP			0.236			0.220
≤ 20mm	127 (59.9)	463 (55.4)		60.9	55.1	
> 20mm	85 (40.1)	373 (44.6)		39.1	44.9	
Lymphovascular space invasion			0.783			0.355
No	118 (55.7)	457 (54.7)		52.1	54.7	
Yes	77 (36.3)	285 (34.1)		40.0	34.8	
Not reported	17 (8.0)	94 (11.2)		7.9	10.5	
Parametrial space invasion			0.105			0.880
No	209 (98.6)	803 (96.1)		97.2	96.6	
Yes	2 (0.9)	24 (2.9)		2.2	2.5	
Not reported	1 (0.5)	9 (1.1)		0.5	1.0	
Vaginal space invasion			0.892			0.878
No	204 (96.2)	800 (95.7)		94.6	95.9	
Yes	5 (2.4)	21 (2.5)		2.7	2.5	
Not reported	3 (1.4)	15 (1.8)		2.7	1.6	
Uterine invasion			0.823			0.376
No	191 (90.1)	763 (91.3)		86.6	91.2	
Yes	15 (7.1)	56 (6.7)		8.7	6.5	
Not reported	6 (2.8)	17 (2.0)		4.7	2.3	
Affected margins			0.452			0.726
No	201 (94.8)	777 (92.9)		95.4	93.0	
Yes	8 (3.8)	49 (5.9)		2.4	5.9	
Not reported	3 (1.4)	9 (1.1)		2.2	1.0	
Surgical complications			0.360			0.205
No	193 (91.0)	740 (88.5)		92.8	88.6	
Yes	17 (8.0)	84 (10.0)		6.9	10.0	
Not reported	2 (0.9)	12 (1.4)		0.4	1.4	
Surgical approach			0.000			0.766
Laparoscopic approach	159 (75.0)	316 (37.8)		46.7	45.3	
Open approach	53 (25.0)	520 (62.2)		53.3	54.7	
Type of radical hysterectomy			0.869			0.322
Type B	245 (30.1)	62 (30.7)		30.4	26.5	
Type C	569 (69.9)	140 (69.3)		66.8	71.5	
Not reported	10 (4.7)	22 (2.6)		2.0	2.8	
Conization			0.010			0.816
No	149 (70.3)	508 (60.8)		63.8	62.7	
Yes	63 (29.7)	328 (39.2)		36.2	37.3	
Use of protective maneuvers			0.000			0.859
No	617 (73.8)	97 (45.8)		69.4	64.5	
Closure of vagina over tumor	162 (19.4)	110 (51.9)		22.8	33.5	
Specimen extracted within a bag	57 (6.8)	5 (2.4)		7.7	2.0	
Macroscopic appearance			0.093			0.681
Exophytic	93 (43.9)	357 (42.7)		41.7	43.1	
Endophytic ulcerative	31 (14.6)	175 (20.9)		14.7	21.4	
Endophytic barrel shaped	21 (9.9)	62 (7.4)		7.6	7.2	
Histology in AP sample			0.393			0.226
Squamous carcinoma	141 (66.5)	573 (68.5)		71.8	68.1	
Adenocarcinoma	66 (31.1)	231 (27.6)		26.5	28.2	
Adenosquamous carcinoma	5 (2.4)	32 (3.8)		1.7	3.7	
Grade of diferentiation			0.794			0.820
Grade I	31 (14.6)	131 (15.7)		13.3	15.7	
Grade II	87 (41.0)	358 (42.8)		43.9	43.4	
Grade III	64 (30.2)	236 (28.2)		27.0	28.7	

The weighted cohort is adjusted for surgical approach, use of uterine manipulator, lymphovascular space invasion, parametrial space invasion, and conization using the IPW method

*SNB* sentinel node biopsy, *SD* standard deviation, *BMI* body mass index

Out of the 1048 women included in this study, 14.0% had positive nodal status determined by SNB + lymphadenectomy compared with 10.7% in the lymphadenectomy group. Nevertheless, this difference of 3.3% (95% CI −2.8% to 9.2%) did not prove to be statistically significant (*p* = 0.30).

Results from the primary endpoint of the analysis indicated that women whose lymphatic node invasion was determined using lymphadenectomy were more likely to receive adjuvant treatment than those evaluated using SNB + lymphadenectomy in the weighted cohort [44.1% versus 33.8%, respectively (Table 2)]. Precisely, we found a difference in the adjusted proportion of 10.9% (95% CI 2.14–19.6%, *p* = 0.015) regardless of nodal status. That difference was greater in the analyses restricted to women with negative nodal status (38.1% in the lymphadenectomy group versus 25.1% in the SNB + lymphadenectomy group). This difference of 13.7% (95% CI 5.0–22.4%, *p* = 0.002) was statistically significant. Stratified analysis in this group of women showed that this difference in adjuvant treatment was even more evident in women with positive Sedlis criteria (Table 2). Among those, 74.5% of women in the lymphadenectomy group versus 43.3% of women in the SNB + lymphadenectomy group received adjuvant therapy (difference 31.2%, 95% CI 7.3–55.1%, *p* = 0.001). Eight of the women in the study (0.8%) had not received lymphadenectomy or the information regarding lymphadenectomy was insufficient. Sensitivity analysis excluding these women did not affect the results (data not shown).

**Table 2:** Proportion of women in the SUCCOR cohort that underwent adjuvant therapy by method of lymph node diagnosis

	Lymphadenectomy	SNB + lymphadenectomy	Adjusted difference* (95% CI)	*p*
*Proportion of women that underwent adjuvant therapy*				
Overall sample	44.1%	33.8%	10.9% (2.14% to 19.6%)	0.015
*Proportion of women that underwent adjuvant therapy in restricted analysis*				
All women without nodal invasion	38.8%	25.1%	13.7% (5.0% to 22.4%)	0.002
Women without nodal invasion and positive Sedlis criteria	74.5%	43.3%	31.2% (7.3% to 55.1%)	0.011
Women without nodal invasion and negative Sedlis criteria	29.3%	22.8%	6.57% (− 3.75% to 16.9%)	0.212

^*^Adjusted for surgical approach, use of uterine manipulator, lymphovascular space invasion, parametrial space invasion and conization using the IPW method. *CI* confidence interval, *SNB* sentinel node biopsy

**Fig. 2:**
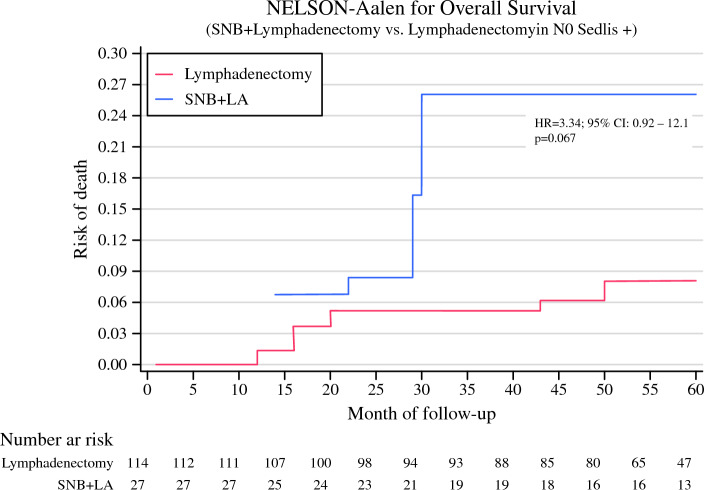
Nelson–Aalen curve for overall survival of women in the SUCCOR cohort with negative nodal status and positive Sedlis criteria, based on method of diagnosis of lymph node metastasis (lymphadenectomy versus SNB+ lymphadenectomy), adjusted for surgical approach, use of uterine manipulator, lymphovascular space invasion, parametrial space invasion, and conization using the IPW method. *SNB* sentinel node biopsy, *LA* lymphadenectomy, *HR* hazard ratio, *CI* confidence interval

In the analyses restricted to women with negative nodal status and positive Sedlis criteria, we observed a greater incidence rate of mortality in the group that received SNB + lymphadenectomy compared with the lymphadenectomy group in the weighted cohort (Fig. 2).

After additionally adjusting for adjuvant treatment (Table 3), the mortality rate was still greater in the group of women who underwent SNB + lymphadenectomy (4.76 per 1000 person/month) than in the lymphadenectomy group (1.41 per 1000 person/month), but the adjusted difference was non-significant (difference 3.35%, 95% CI −1.50 to 7.69 per 1000 person/month). Nevertheless, this difference translated to a 3-times greater hazard of death in the SNB+ lymphadenectomy group compared with the lymphadenectomy group (HR 3.49, 95% CI 1.04–11.7, *p* = 0.042) independently of the treatment.

**Table 3:** Hazard ratio (HR) and 95% confidence interval (CI) for overall survival of women in the SUCCOR cohort with negative nodal status and positive Sedlis criteria, associated with method of lymph node invasion diagnosis, additionally adjusted for adjuvant treatment

	Lymphadenectomy	SNB + lymphadenectomy	Adjusted margins (95% CI)*		
Percentage of events	6.1 %	22.2 %			
Time at risk (person/months)	5,531	1,291			
Incidence rate (per 1000 person/month)	1.41	4.76	3.35 (− 1.50 to 7.69)		

	HR	95% CI	HR	95% CI	*p*
Overall survival	1.00	Ref.	3.49	1.04–11.7	0.042

^*^Adjusted for surgical approach, use of uterine manipulator, lymphovascular space invasion, parametrial space invasion and conization using the IPW method. *SNB* sentinel node biopsy, *CI* confidence interval, *HR* hazard ratio

**Fig. 3:**
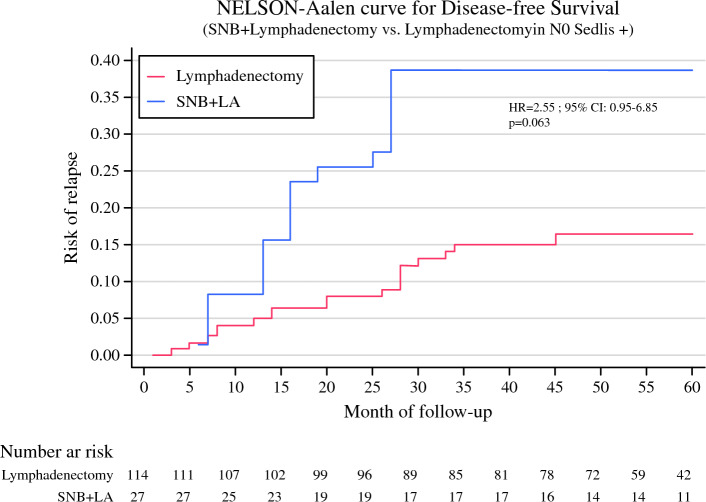
Nelson–Aalen curve for disease free survival of women in the SUCCOR cohort with negative nodal status and positive Sedlis criteria, based on method of diagnosis of lymph node metastasis (SNB versus lymphadenectomy), adjusted for surgical approach, use of uterine manipulator, lymphovascular space invasion, parametrial space invasion and conization using the IPW method. *SNB* sentinel node biopsy, *LA* lymphadenectomy, *HR* hazard ratio, *CI* confidence interval

Regarding disease free survival in the group of women with negative nodal status and positive Sedlis criteria, a greater incidence rate of relapse was observed in the group that underwent SNB + lymphadenectomy than in the lymphadenectomy group (Fig. 3).

After additionally adjusting for treatment, we also observed a greater incidence rate of relapse in women who underwent SNB + lymphadenectomy (7.72 per 1000 person/month) than in those who underwent lymphadenectomy (3.03 per 1000 person/month) but the adjusted proportion did not reach statistical significance (difference 4.69%, 95% CI − 1.57 to 9.23 per 1000 person/month). However, this difference indicated that women in our sample in the SNB + lymphadenectomy group had 2.5-fold greater hazard of relapse than those in the group diagnosed using only lymphadenectomy (HR 2.49, 95% CI 0.98–6.33, *p* = 0.056) independently of the treatment (Table 4).

**Table 4:** Hazard ratio (HR) and 95% confidence interval (CI) for disease free survival of women in the SUCCOR cohort with negative nodal status and positive Sedlis criteria, associated with method of lymph node invasion diagnosis, additionally adjusted for adjuvant treatment

	Lymphadenectomy	SNB + lymphadenectomy	Adjusted margins (95% CI)*		
Percentage of events	14.0 %	33.3 %			
Time at risk (person/months)	5,259	1,135			
Incidence rate (per 1000 persons/month)	3.03	7.72	4.69 (− 1.50 to 9.23)		

	HR	95% CI	HR	95% CI	*p*
Disease free survival	1.00	Ref.	2.49	0.98–6.33	0.056

^*^Adjusted for surgical approach, use of uterine manipulator, lymphovascular space invasion, parametrial space invasion and conization using the IPW method. *SNB* sentinel node biopsy, *CI* confidence interval, *HR* hazard ratio

## Discussion

In this study we presented the differences in treatment with adjuvant therapy of women with a FIGO 2009 stage IB1 cervical cancer who underwent a radical hysterectomy, according to the method used to diagnose lymph node status. In the analysis restricted to women with negative nodal invasion, the proportion of women that received adjuvant therapy was significantly higher in the group of women who underwent a lymphadenectomy alone than in those who underwent a SNB + lymphadenectomy. Interestingly, this difference occurred particularly in women with positive Sedlis criteria. To our knowledge, this is the first study reporting a significant difference in the postoperative management of women according to the type of diagnostic method used for lymph node analysis. Despite the need to replicate our results, we think they hold great importance, given that they identified a group of women that might be undertreated. Future studies with larger sample sizes are needed to elucidate whether the observed difference has an impact on these patients’ disease-free and overall survival.

As of now, European clinical guidelines support the consideration of adjuvant therapy in women with stage IB1 cervical cancer, especially if there is a combination of risk factors such as lymph node involvement, lymphovascular space invasion, large tumor size, and deep stromal invasion.^9^ FIGO guidelines also recommend postoperative radiotherapy in the patients with intermediate risk factors, as they are associated with a greater risk of relapse.^5^ Specifically, patients are considered intermediate risk if they present at least two of the following characteristics: deep stromal invasion (>1/3), large tumor diameter (2 cm or 4 cm^6, 7, 10, 11^), and lymphovascular space invasion. A retrospective study carried out by the Gynecologic Oncology Group corroborated that these clinical characteristics have been associated with greater risk of relapse.^10^ Nevertheless, clinical guidelines also warn the possibility that other clinical factors, different from the classical ones, may also be associated with a greater risk of recurrence: from tumor histology to tumoral implication of surgical margins.^11, 12^ Despite this evidence, to our knowledge, the diagnostic method used for lymph node analysis is not an indicator of neither the risk of relapse nor the need of adjuvant therapy.

Conversely, FIGO guidelines state that women without high or intermediate risk factors should not be considered for postoperative radiotherapy.^5^ Still, in our study, 29.5% of node-negative women who did not meet intermediate-risk criteria in the lymphadenectomy group did receive adjuvant treatment. A more detailed analysis of each case would be necessary to determine whether there was an objective indication for adjuvant therapy or whether these women were in fact overtreated. However, all in all, these findings reflect that the indication of adjuvant therapy has not been standardized and could benefit from a protocol that unifies its indication.

In addition to its indication, the evidence on the efficacy of adjuvant therapy is also inconclusive. Until now, it was believed that adjuvant therapy was effective in prolonging disease-free survival at the expense of a greater morbidity.^6^ Nevertheless, Cibula et al*.* published a retrospective trial in 2018 that found no significant differences, neither in recurrence pattern nor overall survival between women with intermediate risk cervical cancer treated exclusively with surgery versus surgery + adjuvant radiotherapy.^13^ Nakamura et al. reiterated these findings and further associated radiotherapy with an increased risk of lymphedema.^14^ More recently, in 2022, a metaanalysis by Gómez-Hidalgo et al. did not find significant differences in the disease-free and overall survival of patients who were or were not treated with adjuvant therapy, in the studies included.^2^ Nevertheless, important limitations hamper the findings of the last study: most importantly the presence of residual confounding, as the studies were retrospective, and the uneven distribution of risk factors, which favored the group without adjuvant treatment. In our analyses, the hazard ratio of recurrence and of death from the disease were adjusted for the received treatment (Tables 3, 4). Therefore, the differences in disease-free survival and overall survival observed between groups cannot be attributed to the differences in adjuvant treatment. Indeed, determining the efficacy of adjuvant therapy is a challenge beyond the scope of this study. Evidence drawn from the CERVANTES trial will probably help clarify this controverted subject to better the treatment of patients.^15^

In the scheme of risk-benefit attributed to the use of adjuvant radiotherapy, there are several complications that need to be closely considered from the acute inflammatory consequences that affect mostly the gastrointestinal tract, to the chronic ischemic effect on tissues that arises from pelvic radiation.^2, 16^ Recent cohort studies suggest that 10–20% of patients that undergo pelvic radiotherapy develop gastrointestinal toxicity during a 10-year period.^17^ Effects range from radiation enteritis, ileitis, and proctitis that manifest as diarrhea, bleeding, and urgency; or more severely in the chronic stages, intestinal necrosis.^16, 18^ Other adverse effects include hematologic toxicity (leukopenia and lymphopenia),^19^ and more anecdotally, cystitis and vaginal stricture. Hence, overall, the impact on quality of life is significant, and must be heavily considered.

When considering the differences in the allocation of adjuvant therapy between both groups, one possible explanation for the results is that physicians find a negative result more reliable if it is determined using SNB. There is a tendency to believe that lymphadenectomies yield more false negatives than sentinel node biopsies. In fact, studies show that SNB has a sensitivity of 88%, with a negative likelihood ratio of 16% in tumors ≤ 2 cm, allowing detection of an additional 8% of cases that would go undetected with only hematoxylin and eosin (H&E) staining.^20^ However, in the weighted cohort we found that the proportion of women with nodal invasion was similar in both groups. For this reason, we believe that beyond the increase in diagnostic potential that the SNB holds, a false sense of security has been attributed to this technique, that has led to an overconfidence in negative results to the point of ignoring clinical criteria. Nevertheless, validation studies are required to compare diagnostic yield of both techniques so that this belief can be confirmed or discarded.

Likewise, previous studies have suggested a therapeutic benefit from lymphadenectomy, as this technique may irradicate residual microscopic illness.^21^ It is important to note that our study compared women who had undergone SNB+ lymphadenectomy with women who had undergone lymphadenectomy alone, so our findings cannot answer that question. More than 99% of the women in our study had undergone a lymphadenectomy, and sensitivity analysis excluding those who had not did not affect the results. Therefore, the possible therapeutic effect of lymphadenectomy cannot be used as an explanation for the between groups differences observed in this study.

The LACC trial showed that women intervened through laparoscopy had a worse prognosis than those who underwent a laparotomy.^22^ In our study, surgical approach was homogeneous in both groups after IPW, meaning that this factor could not be held accountable for the results we found. Moreover, the SUCCOR study shed light on the role of the uterine manipulator in a worse overall outcome and the favourable effect of the use of protective maneuvers.^23^ Still, both groups were similar in these regards. Along with this, the type of radical hysterectomy was also balanced between both groups in the weighted cohort and, therefore, could not be considered as an explanation for the observed differences in prognosis.

This study has several strengths. Firstly, we managed a large sample size, which provided sufficient statistical power to draw significant results. Secondly, we used inverse probability weighting to adjust for potential confounders and obtained a weighed cohort in which the comparison groups were similar in sociodemographic and clinical characteristics. Thirdly, this is a multicentric study, with the participation of 126 institutions from 29 European countries, which offers a greater diversity of data and provides greater external validity to our results. On the other hand, we must acknowledge some limitations to our study. Firstly, the retrospective design of the study allows for misclassification bias. However, data was extracted from medical records, most of them computerized, which allows us to assume their validity. Secondly, using IPW artificially standardizes a pseudopopulation based on the variables included in the propensity score. Thirdly, the sample size in the group with negative nodal invasion and positive Sedlis criteria was smaller than ideal for adequate statistical power. Since our results were still significant, we encourage further studies with a larger sample size to replicate our findings. Lastly, since this is an observational study, we cannot discard the possibility of residual confounding. However, in cases where clinical trials are not feasible, the best possible evidence is obtained from observational studies with adequate follow-up of participants and close control of confounding.^24^

In conclusion, in the SUCCOR cohort, the proportion of women with intermediate-risk factors and nodal involvement was similar in both the lymphadenectomy and SNB + lymphadenectomy groups. Nevertheless, adjuvant therapy was administered more frequently in the lymphadenectomy group. Regarding prognosis, women with negative nodal status and positive Sedlis criteria who underwent lymphadenectomy presented better overall survival and a marginally better disease-free survival than those who underwent SNB + lymphadenectomy independently of the treatment received. In this scenario, we do believe that it is necessary to review the recommendations of clinical practice guidelines regarding adjuvant treatment of women with FIGO 2009 IB1 cervical cancer and their implementation.

## Supplementary Information

Below is the link to the electronic supplementary material.

Supplementary file1 (PDF 25 kb)
