# Supplementary material for: SUCCOR Nodes: May Sentinel Node Biopsy Determine the Need for Adjuvant Treatment?
Source: Ann Surg Oncol. 2023 May 19;30(8):4975–85. doi: 10.1245/s10434-023-13529-w (PMC10319697; doi:10.1245/s10434-023-13529-w)
Supplement: Supplementary file 1 — Supplementary file1 (PDF 25 kb) [file 10434_2023_13529_MOESM1_ESM.pdf]

Supplementary table 1

Main inclusion criteria

1. Underwent a radical hysterectomy in a European Institution for stage IB1 cervical cancer (FIGO 2009), from January 1, 2013 to December 31, 2014.
2. Patients age 18 years' old or older and
3. Histology must be either squamous cell carcinoma, adenosquamous carcinoma, or adenocarcinoma.
4. Patient must have received a preoperative pelvic MRI confirming
    a. a tumor diameter less or equal than 4 cm
    b. with no parametrial invasion and
5. Patient must have received a preoperative CT scan, MRI, or PET-CT without extracervical metastatic disease.
6. The operative report had to describe either a Type B–C radical hysterectomy with a bilateral pelvic lymphadenectomy, including at least a total of 10 pelvic nodes.
7. There had to be documentation of tumor size, margins, and nodal status.

Main exclusion cirteria

1. Any other histological type was diagnosed;
2. Tumor size larger than 4cm;
3. Evidence of past history of any invasive tumor, previous chemotherapy or radiation, suspicious lymph nodes, or metastatic disease on preoperative imaging;
4. Conversion from minimally invasive surgery to laparotomy;
5. Missing data on follow-up or adjuvant therapy;
6. Less than 10 pelvic lymph node count.
7. Missing data on performance of SNB+lymphadenectomy or lymphadenectomy

Supplementary table 2

Evaluation of compliance of Sedlis criteria

LVSI	Stromal invasion (deep 1/3)	Tumor diameter ≥ 2 cm	SEDLIS CRITERIA
+	deep	-	Yes
+	middle	+	Yes
